# Ubi-SCAPE Enables Deep Exploration of the Poly-Ubiquitylome

**DOI:** 10.1021/acs.jproteome.6c00151

**Published:** 2026-10-13

**Authors:** Harvey E. Johnston, Andrew M. Frey, Gaurav Barve, Sam Carling, Matthias Trost, Rahul S. Samant

**Affiliations:** † Signalling Programme, 2150 Babraham Institute, Cambridge CB22 3AT, U.K.; ‡ Biosciences Institute, 5994 Newcastle University, Newcastle-upon-Tyne NE2 4HH, U.K.; § Translational and Clinical Research Institute, Newcastle University, Newcastle-upon-Tyne NE2 4HH, U.K.; ∥ School of Biology, University of Manchester, Manchester M13 9PL, U.K.

**Keywords:** ubiquitin, poly-ubiquitin, affinity purification, post-translational modification, PTM, Lys-ε-GlyGly.

## Abstract

Post-translational modification with chains of the 76-amino acid protein ubiquitin (“poly-ubiquitylation”) confers diverse fates to the targeted protein and non-protein substrates, including degradation, intracellular trafficking, and signal transduction. Despite being one of the most frequent modifications, the complexity of poly-ubiquitin chains adds methodological challenges to their characterization. Trypsin-resistant tandem ubiquitin-binding entities (trTUBEs), engineered from natural ubiquitin-binding domains, can capture intact poly-ubiquitylated material with high cumulative avidity. However, such approaches have suffered from considerable co-eluting contaminants in proteomics applications. Here, we introduce an optimized trTUBE-based method for poly-ubiquitylated proteome purification, drastically depleting non-ubiquitylated protein contaminants and mono-ubiquitylated proteins. The method, termed ubiquitylomics by stringent, cleavable, affinity-based proteome extraction (Ubi-SCAPE), offers a streamlined and reproducible (median *R* ^2^ > 0.98; CV < 8%) means of characterizing the poly-ubiquitylome. Over 7800 poly-ubiquitylated proteins and 8500 ubiquitin-modified peptides (diGly) were quantified with Ubi-SCAPE at a throughput of 40 samples per day, as well as over 6000 proteins from 100 μg of unstressed cell lysate. Upon acute stress by heat-shock, we identified 2700 proteins and 8000 diGly peptides with increased poly-ubiquitylation, offering similar biological insights as far more material-, labor-, and cost-intensive peptide-based ubiquitin enrichment methods would provide. Ubi-SCAPE therefore provides a simple and effective means of comprehensively quantifying a selectively enriched poly-ubiquitylome.

## Graphical abstract

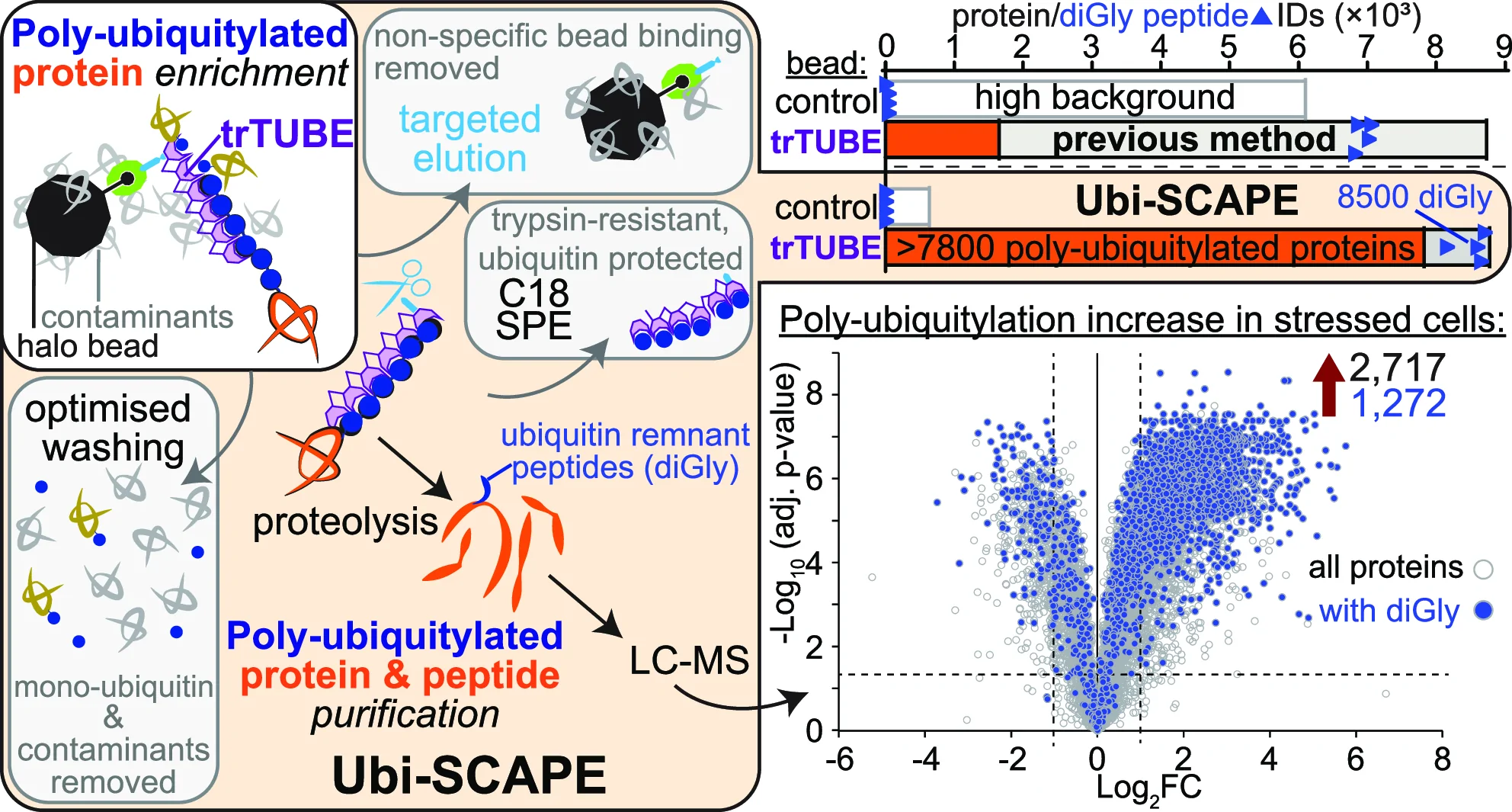

## Introduction

Ubiquitin is a highly conserved and abundant post-translational modifier throughout the proteome of eukaryotic organisms.^1^ Although post-translational modification (PTM) of substrates with one or more ubiquitin units is most commonly associated with targeting them for degradation, “ubiquitylation” has highly diverse roles and potentially represents the most frequent posttranslational modification in the human proteome.^2,3^ Comprehensive identification and quantification of ubiquitylated proteins, therefore, promise substantial insights into cellular pathways and states such as protein misfolding, degradation, and signaling.^4,5^ Rather than representing a uniform modification, ubiquitylation commonly exists as a range of mono-, multi-mono-, or poly-ubiquitin chains on the original substrate. Like other critical PTMs, the conservation and frequency of ubiquitylation across eukaryotes reinforce a universal role across biological processes. Despite this biological importance, tools for its characterization remain relatively limited and infrequently applied.

Current gold-standard approaches to quantify the ubiquitin-modified proteome (“ubiquitylomics”) involve the enrichment of peptides bearing the “diGly” ubiquitin-remnant fingerprint after tryptic digestion, using proprietary antibodies raised against that motif.^2,6–9^ Although this approach is effective for isolating such peptides, it is resource-intensive, requires large amounts of starting material and multiple specialized steps, and cannot distinguish between mono-, poly-, and some ubiquitin-like modifications.^6^ Other tools for the isolation of all free ubiquitin and ubiquitylated proteins have also been developed;^10–18^ the complexity and diversity of poly-ubiquitylation, however, have remained challenging to characterize.

TUBEs (tandem ubiquitin-binding entities) were developed for this purpose,^19^ employing a string of ubiquitin-binding domains to capture ubiquitin chains and potentially some highly multi-mono-ubiquitylated proteins, through high-avidity interactions. Trypsin-resistant TUBEs (trTUBEs) represented an additional improvement, protecting both the TUBE and the bound ubiquitin from tryptic digestion during bottom-up proteomics workflows, thereby reducing the signal from these two major “contaminants” and improving the depth of coverage for the ubiquitin-modified proteome itself.^11^

Although TUBEs and trTUBEs have been employed in proteomics applications,^11,20–24^ their uptake for poly-ubiquitylomics has been limited, perhaps due to the risk of contamination from abundant or co-precipitating proteins that were not themselves poly-ubiquitylated—a challenge for many high-sensitivity proteomics affinity purification workflows.^25^

Here, we present Ubi-SCAPE (ubiquitylomics by stringent, cleavable, affinity-based proteome extraction), a trTUBE-based poly-ubiquitylomics pipeline that yields unprecedented coverage, reproducibility, and specificity, and provides clear improvements over existing ubiquitin-enrichment approaches.

## Materials and Methods

### Materials and Reagents

All key materials and reagents are listed in the key resource table (Table S5).

### Construction of pET28a HaloTag–TEVc–Empty and HaloTag–TEVc–6 × trTUBE

To construct pET28a HaloTag–TEVc–empty (Addgene #254152), a fragment containing the self-labeling HaloTag, the tobacco etch virus protease (TEVp) cleavage site ENFLQG (denoted “TEVc” here), and a multiple-cloning-site (MCS), from pET28a HaloTag–TEVc–[MCS] (DU23222, MRC-PPU Reagents & Services), was PCR-amplified with the custom primers 108-BlpI-F1 (ATTATGCTTAGCGGCAGCAGCCATC) and pET28a-BlpI-R1 (TGCTAGTTATTGCTCAGCGGTGGCA), followed by BlpI digest and ligation into the optimized pET28a T7pCONS TIR-2 sfGFP (gift from Daniel Daley, Addgene #154464).^26^ To construct pET28a HaloTag–TEVc–6×trTUBE (Addgene #254153), the 6×trTUBE insert from pRSET–6×TR-TUBE (gift from Yasushi Saeki, Addgene #110313)^18^ was double-digested with EcoRV + DraIII and ligated into Eco53kI + DraIII double-digested pET28a HaloTag–TEVc–empty. To generate an alternative version of the construct with an additional 6×His tag followed by a thrombin cleavage site for affinity purification (“pET28a 6×His–HaloTag–TEVc–6×trTUBE”; Addgene #254156), the 6×His–HaloTag fragment from pET28a 6×His–HaloTag–Nrf2 (gift from Yimon Aye, Addgene #62455) was double-digested with XhoI + XbaI and ligated into similarly digested pET28a HaloTag–TEVc–6×trTUBE. pET28a 6×His–HaloTag–TEVc–4×trTUBE (Addgene #254154) and pET28a 6×His–HaloTag–TEVc–4×trTUBE_mut_ (Addgene #254155) were constructed by DraIII + *Bgl*II sequential digest of pET28a 6×His–TEVc–HaloTag–4×trTUBE (MRC-PPU Reagents & Services, #DU58810) or pET28a 6×His–TEVc–HaloTag–4×trTUBE_mut_ (MRC-PPU Reagents & Services, #DU58829) (both gifts from Yu-Chiang Lai, U. Birmingham) and ligation into similarly digested pET28a 6×His–HaloTag–TEVc–6×trTUBE.

All cloning and propagation were performed in subcloning-efficiency competent DH5-α *Escherichia coli* (Thermo Fisher Scientific; Cat# 18265017), and all enzymes, buffers, and kits for cloning were from New England Biolabs. All plasmids were verified by whole-plasmid sequencing (PlasmidSaurus).

### trTUBE Expression and Conjugation to HaloTag Magnetic Beads

Rossetta 2­(DE3)-competent *E. coli* (Novagen/Sigma; Cat# 71397) transformed with one of the pET28a HaloTag–TEVc–6×trTUBE, pET28a HaloTag–TEVc–empty, pET28a 6×His–HaloTag–TEVc–4×trTUBE, or pET28a 6×His–HaloTag–TEVc–4×trTUBE_mut_ plasmids was cultured at 37 °C for 18 h to OD_600_ ∼0.7, adjusted to 1 mM IPTG (Melford Laboratories, Cat# B1008) to induce expression, and incubated for 24 h at 16 °C. *E. coli* was pelleted at 7000*g* for 10 min at 4 °C. After supernatant aspiration, the bacteria were lysed with three pellet volumes (w/v) of bacterial lysis buffer (50 mM Tris HCl, pH 7.5, 150 mM NaCl, 1% Triton X-100, 20 mM TCEP (tris­(2-carboxyethyl)­phosphine), 1× complete protease inhibitors, and 0.2 mM PMSF (phenylmethylsulfonyl fluoride)) with sonication on ice for 2 min total at 20 W (10 s intervals) using a non-microtip probe (Covaris). Lysates were cleared by centrifugation for 30 min at 16,000*g* at 4 °C, and the supernatant was snap-frozen in liquid nitrogen and stored at −70 °C as 200 μL aliquots.

Magne-HaloTag bead (Promega, Cat# G7281) saturation with HaloTag–TEVc–6×trTUBE protein was determined (Figure S1) as approximately 140 μL of *E. coli* lysate as prepared above per 1 mL of 20% Magne-HaloTag beads. To accommodate for batch variability, 200 μL of *E. coli* lysate was used per 1 mL for all preparations. Magne-HaloTag beads were washed three times with 1 mL of IP buffer (50 mM Tris–HCl, pH 7.5, 150 mM NaCl, 1% IGEPAL CA-630) and incubated with 200 μL of *E. coli* lysate for 2 h at room temperature with end-over-end rotation at 20 rpm. Beads were then washed three times with 1 mL of trTUBE wash buffer (defined below), three times with IP buffer, and stored at 4 °C as a 2% slurry.

### Cell Culture and Heat-Shock

HEK293 cells were grown in DMEM (Gibco; Cat# 41965039) supplemented with 10% heat-inactivated HyClone characterized fetal bovine serum (Gibco; Cat# SH30071.02HI), 100 U/mL penicillin–streptomycin (Gibco; Cat# 15140122), 2 mM l-glutamine (Gibco; Cat# 25030081), and 1× nonessential amino-acids (Gibco; Cat# 11140050), in a humidified incubator set at 37 °C with 5% CO_2_. Cells plated from a single pool of cells and grown to ∼80% confluency were either left at 37 °C (‘unstressed’) or incubated at 44 °C for 2 h (“heat-shock”, HS). Media were carefully removed from the culture dishes by aspiration, and cells were gently resuspended in 10 mL of ice-cold phosphate-buffered saline (PBS)­(Sigma; Cat# P2272) per 15 cm plate, washed three times with 10 mL of PBS per 15 cm plate, pelleted at 300*g*, fully aspirated, and snap-frozen in liquid nitrogen.

### Cell Lysate Preparation

Cell pellets were lysed on ice in four cell-pellet volumes (v/v) of chilled, modified SP3 buffer,^27^ termed “urea SP3 lysis buffer” (50 mM HEPES pH 8.0, 8 M urea, 1% SDS, 1% Triton X-100, 1% IGEPAL CA-630, 1% Tween 20, 1% sodium deoxycholate, 150 mM NaCl, 10 mM TCEP, 1× cOmplete protease inhibitors, EDTA-free, 1 mM PMSF, 10 μM PR-619, 40 mM 2-chloroacetamide, 20 U/μL Pierce Universal Nucleases (Thermo; Cat# 88701)). Lysates were incubated on ice for 15 min and transferred to a thermomixer for 30 min at 25 °C, 800 rpm for reduction, alkylation, and nuclease action. Lysates were cleared at 16,000*g* for 5 min at 4 °C. Protein concentration was estimated by BCA assay (Thermo; Cat# 23225) of peptides generated by SP4 protein cleanup,^28^ with lysates adjusted to 5 μg/μL.

### Poly-Ubiquitylome Purification with trTUBEs

Affinity purifications were performed with 500 μg of BCA-quantified cell lysate (or 100 μg, where specified), typically in 100 μL of lysis buffer, diluted in 1700 μL of IP buffer, with 200 μL of 2% HaloTag bead:IP buffer slurry to give a final lysate:IP buffer dilution of 1:20, and incubated for 18 h at 4 °C with end-over-end rotation at 20 rpm. Replicates were defined as full technical repeats of independent lysate:bead incubations. Beads were gathered by a magnet, aspirated, and washed three times (each with 30× manual end-over-end inversions over ∼1 min) with 1 mL of either a “typical” wash buffer (IP buffer adjusted to 500 mM NaCl), representative of wash buffers previously used for TUBE enrichments,^11,20–24^ or an optimized “trTUBE wash buffer” (IP buffer supplemented with 5% SP3 buffer, 2 M urea, 1 M NaCl). Beads were subsequently washed three times with 1 mL of 50 mM HEPES pH 8.0. For the final wash, beads were transferred to a new 1.5 mL Protein LoBind tube (Eppendorf) and fully aspirated with the aid of brief centrifugation (2000*g*, 15 s). Tubes were placed on ice and 1 μL (10 U) of tobacco etch virus protease (TEVp) (New England Biolabs, MA USA; Cat# P8112) in 20 μL of 50 mM HEPES pH 8.0 was added and incubated for 5 min prior to transferring to a thermomixer for 2 h at 30 °C and 1000 rpm to elute poly-ubiquitylated proteins from the beads. Beads were gathered by a magnet, with the supernatant containing complexes of TEVp-eluted, trTUBE-bound poly-ubiquitylated proteins.

### Recombinant Poly-Ubiquitin Capture with trTUBEs

100 ng of each human recombinant di-ubiquitin chain (K6-, K11-, K29-, K33-, K48-, and K63-linked) (South Bay Bio; SBB-UP0060, 0063, 0077, 0066, 0069, and 0072, respectively), tetra-ubiquitin chain (K6-, K11-, K29-, K33-, K48-, and K63-linked) (South Bay Bio; SBB-UP0061, 0064, 0078, 0067, 0070, and 0073, respectively), linear M1-linked poly-ubiquitin chain (Ub2–7) (Enzo; BML-UW1010), and mono-ubiquitin (Sigma; Cat# U5507) was incubated with trTUBE as described above. Elutions were performed either sequentially, as detailed in each figure, using 15 μL of each potential elution condition for 2 min at 4 °C and 800 rpm or in 1× SDS–PAGE loading buffer (25 mM Tris–HCl pH 6.8, 10% glycerol, 1% SDS, 0.05% (w/v) Orange G) for 10 min at 75 °C and 800 rpm in a thermomixer.

### SDS–PAGE, Staining, and Immunoblotting

Protein solutions in 1× SDS–PAGE loading buffer were run on 10% or 15% tris-glycine-SDS polyacrylamide gels and stained with Coomassie dye for total protein or with mouse anti-HaloTag (Promega; Cat# G9211) or mouse anti–ubiquitin (LifeSensors, Cat# VU101) primary antibody followed by either DyLight 680 or DyLight 800-conjugated anti–mouse IgG secondary antibody (Cell Signaling Technology; Cat# 5470 or 5257, respectively). Immunoblots were visualized on a LiCor Odyssey CLx Imager (LI-COR Biotech).

### Protein Digestion

TEVp-eluted proteins were added to protein LoBind tubes (Eppendorf) containing 50 ng of trypsin and Lys-C (Promega; Cat# V5073) in 10 μL of 50 mM HEPES pH 8.0 (prepared as a master mix with tubes incubated on ice for 5 min). For on-bead digests, 10 μL of the above master mix was added to the aspirated beads suspended in 20 μL of 50 mM HEPES pH 8.0. Digests were incubated for 2 h at 47 °C on a thermomixer (Eppendorf) at 1000 rpm. Post-digestion, samples were acidified to a final concentration of 1% LC-MS-grade formic acid (Fisher).

### LC–MS Proteomics

Liquid chromatography (LC) was performed using an Evosep One system with a 15 cm Aurora Elite C18 column with an integrated CaptiveSpray emitter (IonOpticks), at 50 °C. Buffer A was 0.1% formic acid in HPLC water, and buffer B was 0.1% formic acid in acetonitrile. Immediately prior to LC-MS, 10 μL (one-third) of the resulting peptides was loaded onto the LC system–specific C18 EvoTips, according to the manufacturer’s instructions, and subjected to the predefined WhisperZoom 40 SPD protocol (where the gradient is 0–35% buffer B, 200 nL/min, for 32.5 min, 40 SPDs are permitted using this method). The Evosep One was used in line with a timsToF-HT mass spectrometer (Bruker), operated in diaPASEF mode. Mass and IM ranges at tims-MS1 were 100–1700 *m*/*z* and 0.75–1.45 1/K0, respectively. MS2 during diaPASEF was performed using 16 variable width IM-*m*/*z* windows across two PASEF ramps (Table S3) designed using py_diAID^29^ as part of an ongoing ubiquitylation study. TIMS accumulation and ramp times were 100 ms with a ramp rate of 9.42 Hz, and the total cycle time was ∼1.8 s. Collision energy was applied in a linear fashion, where ion mobility = 0.6–1.6 1/K0 and collision energy = 20–59 eV.

### LC-MS Data Processing

Raw data were initially processed by DIA-NN (v.1.9.0) using its *in silico* generated spectral library function.^30^ Reference proteome FASTA files included *Homo sapiens* (UP000001414, downloaded from UniProt on 23/01/2023) and *E. coli* (UP000000625, downloaded from UniProt on 25/04/2023) and a common contaminant list with the addition of TEVp and the HaloTag sequences.^25^ In addition, the RPS27A and UBA52 protein reference sequences were truncated to exclude the ubiquitin fusion open reading frame, and the poly-ubiquitin proteins (UBB and UBC) were truncated to di-ubiquitin for ease of annotation. Trypsin specificity with a maximum of two variable modifications and one missed cleavage were permitted per peptide; cysteine carbamidomethylation was set as a fixed modification; and oxidation of methionine and K-GG (diGly-lysine, missed cleavage required) was set as variable modifications. Peptide length and *m*/*z* were 7–30 and 300–1256, respectively, and charge states 2–4 were included. Mass accuracy was fixed to 15 ppm for both MS1 and MS2. Protein and peptide FDR were both set to 1%. Match between runs was used. Normalization was disabled to precisely assess technical effects. All other settings were left as defaults.

### Data Analysis and Bioinformatics

Proteomics data for quantified proteins and diGly remnant peptides are detailed in Tables S1 and S2. A custom R script utilizing R (v4.2.2) and the diann-R package (v1.9) was used to generate MaxLFQ-based intensity tables for protein groups, precursors, and diGly peptides from the main report file (.parquet) generated by DIA-NN. Although the majority of diGly residues were internal, a minority was annotated as the C-terminal. On inspection of these peptides, most of these (823/1010) contained an additional uncleaved lysine residue within five amino acids of the C-terminally annotated diGly site, none of which were observed in any nonbinding control samples; therefore, these were considered to be misannotated and were included in analyses. The MS proteomics data have been deposited to the ProteomeXchange Consortium (http://proteomecentral.proteomexchange.org) via the PRIDE partner repository with the dataset identifier PXD073597.^31^

Confident poly-ubiquitylated proteins were strictly defined as those proteins identified in trTUBE isolations that were not observed for any of the four non-binding bead replicates (HaloTag–TEVc–empty) for a respective technical condition control. This classification was based purely on the presence or absence of protein identification defined by DIA-NN, to avoid an arbitrary quantitative threshold. Notably, trTUBEs may also enrich for multi-mono-ubiquitylated proteins, and for ease of clarity, we have referred to these as poly-ubiquitylated proteins here. Proteins reaching an FDR-adjusted *p* < 0.05 and a fold-change of at least 2-fold upon heat-shock (Log_2_FC > 1) were considered significantly overabundant poly-ubiquitylated proteins. Gene ontology (GO) term enrichment analysis and functional annotation enrichment were performed with Database for Annotation, Visualization, and Integrated Discovery (DAVID)­(v2023q4).^32^ Terms were filtered to include those with Benjamini-adjusted *p* < 0.05. StringDB (v11)^33^ was used to visualize the top 200 significantly increased poly-ubiquitylated proteins in heat-shock. The network was trimmed of unconnected proteins with confidence set to “high” (>0.7), with the specified terms highlighted for proteins annotated, selected based on both term-enrichment significance and number of proteins.^33^

## Results and Discussion

### Establishing a Stringent Poly-Ubiquitylated Proteome Enrichment Pipeline

Like many affinity purifications, TUBE-based enrichment of poly-ubiquitylated proteins from complex, high-dynamic range protein mixtures presents a challenge in distinguishing true substrates from non-specific binding or contamination from abundant or adhesive proteins.^34^ Existing applications have employed relatively cautious binding and washing conditions, with non-ionic detergents and salts as the only means of minimizing native protein:protein and protein:bead interactions.^11,20–24^ By contrast, final elution of bound substrates often employs harsh denaturants, such as strong ionic detergent, heat, or on-bead trypsin proteolysis. We reasoned that the combination of these two factors would compound the presence of highly abundant and adhesive proteins in the final eluate analyzed by LC-MS and thus substantially limit the confidence of true positives observed by TUBE-based poly-ubiquitylomics.

To reduce these concerns, we designed a workflow for poly-ubiquitylated proteome purification via an engineered HaloTag-fused 6×trTUBE construct (i.e., six tandem repeats of the ubiquitin-associated domain from human UBQLN1, with all Lys and Arg residues mutated to Ala),^11^ with a tobacco etch virus protease (TEVp) cleavage site introduced in between the HaloTag and the 6×trTUBE that would offer a specific, targeted means of elution (Figure 1A,B). We next performed a set of experiments to define the ideal binding purification parameters, including magnetic-HaloTag bead saturation, stringent washing, and specific elution conditions (Figures S1–S3). The optimized protocol employed harsh initial lysis to denature protein and dissociate interactors, followed by the minimal dilution required to restore trTUBE:poly-ubiquitin binding. Similar principles were applied to the wash conditions: we defined the harshest buffer conditions that maintained trTUBE:poly-ubiquitin binding, to ensure the lowest possible contamination from non-specific interactors. Building upon earlier evidence that the trTUBE binds longer poly-ubiquitin chains better than shorter chains,^18^ we found that the denaturant tolerance of the poly-ubiquitin:trTUBE interaction was proportional to chain length (Figure 1C). This observation allowed us to select binding and wash conditions that minimized the retention of mono- and di-ubiquitin. Harsher washing conditions promised a greater depletion of high-abundance proteins, such as ribosomes and histones, while maintaining poly-ubiquitin binding. In addition to their high abundance, histone proteins in particular are canonically mono-ubiquitylated (as an epigenetic mark);^35^ therefore, depletion of histones and other abundant mono-ubiquitylated proteins would result in an increased depth of coverage into other, less-abundant substrates.

**Figure 1:**
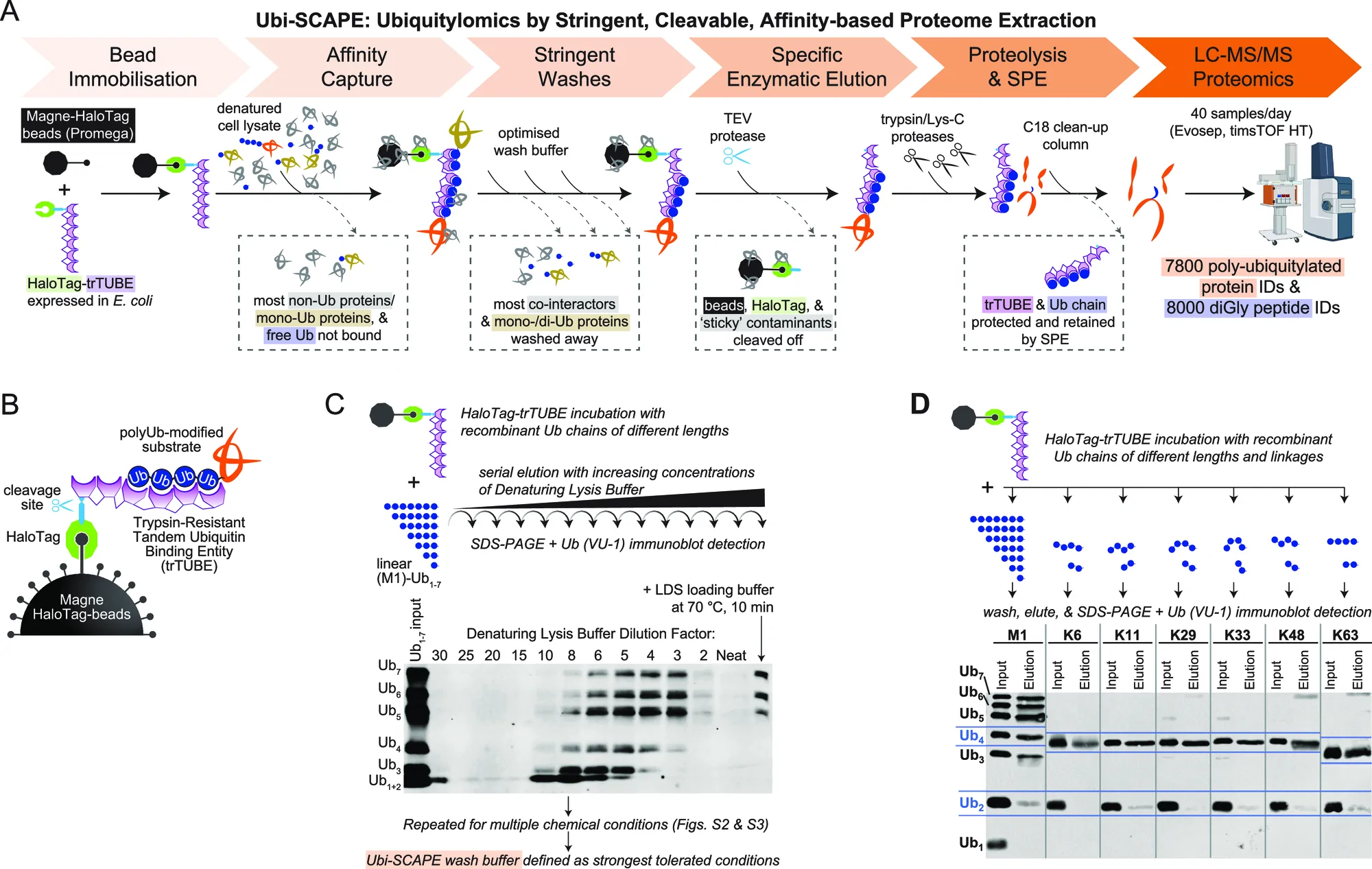
Establishment of the Ubi-SCAPE pipeline for high-purity poly-ubiquitin chain purification. (A) Ubi-SCAPE workflow for purification and quantification of poly-ubiquitylated proteins from diverse biological material, with stringent washes, elution by targeted release, and subsequent characterization by LC-MS-based proteomics of purified, trypsin-digested poly-ubiquitylated protein peptides. (B) Trypsin-resistant tandem ubiquitin-binding entity construct (HaloTag–TEVc–6×trTUBE) conjugated to Magne-HaloTag beads binding to poly-ubiquitylated proteins. (C) Poly-ubiquitin chain length is proportional to a detergent resistance of 6×trTUBE binding, tested using linear (M1-linked) poly-ubiquitin of various lengths (1–7) and iterative incubations to quantify dissociation. (D) trTUBE-based affinity capture is poly-ubiquitin linkage agnostic, excluding mono- and the vast majority of di-ubiquitin. SPE: solid-phase extraction.

The surface area of beads was also a suspected source of contamination, where harsh elution mechanisms could co-dissociate non-specifically bound proteins. Targeted elution of the trTUBE and bound substrate away from the HaloTag beads via TEVp cleavage prior to trypsin digestion presented a promising means of reducing contaminating proteins, in comparison with direct on-bead trypsin digestion.

Our optimized conditions also maintained the linkage non-selective nature of trTUBE (Figure 1D), thus allowing unbiased poly-ubiquitin chain capture.

By employing stringent washing, our *in vitro* work indicated that Ubi-SCAPE would provide more confident purification of the poly-ubiquitylome than previous TUBE-based methods.

### Ubi-SCAPE Purifies the Poly-Ubiquitylome

To evaluate the performance of Ubi-SCAPE vs the most commonly adopted published TUBE-based protocols, a comparative poly-ubiquitylome purification was conducted comparing HaloTag–TEVc–6×trTUBE with a “non-binding” empty HaloTag-only construct (HaloTag–TEVc–empty) to define entirely non-specific protein contamination (Figure 2A). We also evaluated previously described variants of trTUBE with fewer tandem domains, or lower binding capacities, for poly-ubiquitin capture (discussed later). Lysates from HEK293 cells in unstressed or heat-shocked conditions were used to evaluate performance in basal or elevated poly-ubiquitylation settings, respectively. In addition to comparing the trTUBE variants, we also compared our stringent wash conditions with the more typical wash conditions employed previously.^22^

**Figure 2:**
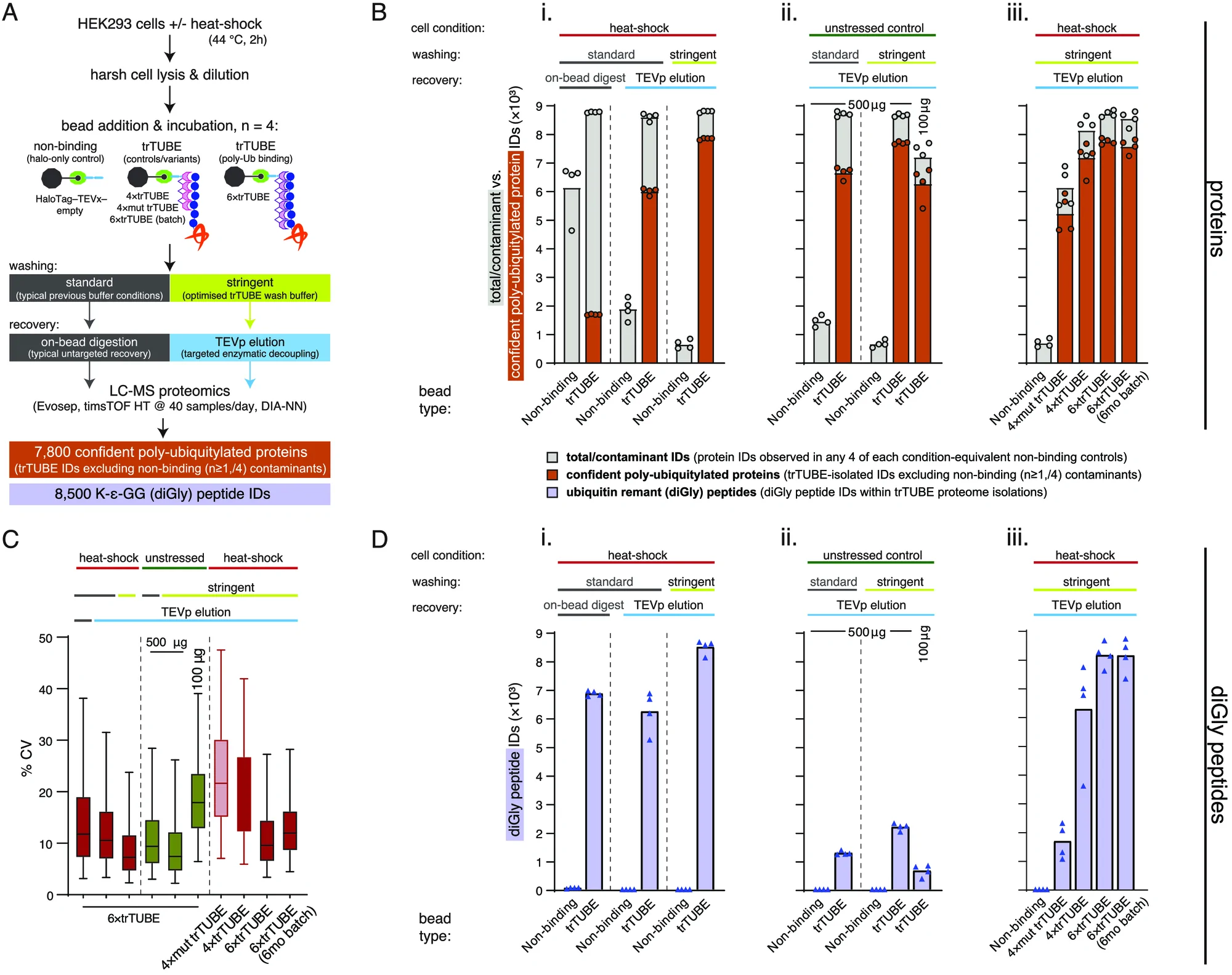
Ubi-SCAPE reproducibly purifies the poly-ubiquitylome with negligible nonspecific binding. (A) Ubi-SCAPE method, incorporating stringent washing and targeted elution, was compared with existing TUBE-based methods employing the equivalent of on-bead digest elution and low-detergent, high-salt washing. For each comparison, trTUBE (HaloTag–TEVc–6×trTUBE) and non-binding (HaloTag–TEVc–empty) bead incubations were performed for 500 μg of lysate, to evaluate poly-ubiquitin capture and the extent of non-specific binding. Both stressed (heat-shock for 2 h at 44 °C) and unstressed cells were evaluated, alongside a more limited-input 100 μg test. Bead variants were also compared, including a binding-deficient mutated HaloTag–TEVc–4×trTUBE_mut_, a shorter HaloTag–TEVc–4×trTUBE, and a batch-stability control for HaloTag–TEVc–6×trTUBE beads stored for 6 months at 4 °C. (B) LC-MS proteomics identifications for evaluations detailed in (A). Confident poly-ubiquitylated proteins (orange) are those IDs observed among total IDs (gray) that were not identified in the corresponding non-binding HaloTag–TEVc–empty (white, deemed as contaminants). (C) Percentage coefficients of variation (% CV) for confident poly-ubiquitylated proteins IDs defined in (B). (D) Lys-ε-GlyGly (diGly) ubiquitin remnant peptide IDs when searching the trTUBE-derived proteome data detailed in (B).

We employed stringent criteria for assigning “true positive” status to consider a protein as confidently poly-ubiquitylated. Only proteins observed eluting exclusively from trTUBE beads, with no observation in any of the four non-binding bead (HaloTag–TEVc–empty) replicates for the equivalent technical conditions, were assigned as confident poly-ubiquitylated proteins (Figure 2B­(i)). Although there were broadly similar numbers of total protein identifications with the HaloTag–TEVc–6×trTUBE across our tested wash and elution parameters, there was a clear difference in the number of proteins identified with the non-binding beads (assigned as contaminants), which had a knock-on effect on the number of poly-ubiquitylated proteins that could be confidently assigned.

As hypothesized, TEVp elution consistently yielded fewer contaminants than on-bead digestion. With standard (non-stringent) washes, we identified up to 6716 non-binding bead-derived contaminant proteins for on-bead digestion; this number fell to 2315 proteins with TEVp-based elution. As a result, the number of proteins that could be considered confidently poly-ubiquitylated increased from 1700 for on-bead digestion to 6700 for TEVp elution. However, standard wash conditions exhibited relatively low poly-ubiquitylated protein reproducibility (Figure 2C) and protein intensities that correlated with the input lysate proteome (*R* ^2^ = 0.5 and 0.23 for on-bead digestion and TEVp elution, respectively), suggesting co-purification of high-abundance contaminants (Figure S4A). By contrast, the Ubi-SCAPE protocol, employing both stringent washing and TEVp-assisted targeted enzymatic release, provided over 7800 confident poly-ubiquitylated proteins, reduced contamination to fewer than 850 proteins, exhibited negligible correlation with the input proteome (*R* ^2^ = 0.046; Figure S4A), and displayed the highest reproducibility (median *R* ^2^ > 0.98, CV < 8%; Figure 2C). We also confirmed that TEVp-assisted release did not lower the overall recovery of proteins from the beads when compared with on-bead digestion (Figure S4B).

The poly-ubiquitylome data were searched for ubiquitin remnant peptides as an independent, supporting metric, considered as validating the successful enrichment of poly-ubiquitylated proteins, as well as detailing the localization of ubiquitylation sites (Figure 2D). Over 8,500 diGly peptides were identified for cells stressed with heat-shock, mapping to over 2,500 proteins—with GCN1, PARP1, XRCC5, HDLBP, GTF2I, FANCI, and UTP20 each exhibiting over 30 sites of ubiquitylation.

Further increasing the denaturation strength of the wash buffer had negligible impact on the recovery of poly-ubiquitylated proteins and diGly peptides (*R* ^2^ > 0.99 and 0.93, respectively; Figure S4C), suggesting that our optimized stringent buffer is sufficient, and also increasing confidence that further non-specifically-bound proteins are only minimally present, if at all.

Even with a much more limited protein input of 100 μg of lysate from unstressed cells, of which one-third was analyzed by LC-MS (Figure 2B­(ii)), we identified between 5411 and 6753 poly-ubiquitylated protein IDs from this limited basal input, further demonstrating the sensitivity of Ubi-SCAPE. Note, however, that far fewer diGly peptides were identified in this experiment (Figure 2D).

To understand further the nature of binding provided by trTUBE-based enrichments, we also evaluated a previously published, shorter HaloTag–4×trTUBE and a binding-deficient HaloTag–4×trTUBE, where each ubiquitin-associated domain had been mutated to lower its affinity for ubiquitin (HaloTag–4×trTUBE_mut_). The 4×trTUBE and 4×trTUBE_mut_ both demonstrated reduced yields, diGly IDs, and reproducibility (Figure 2B­(iii)). Notably, the binding-deficient mutant exhibited a uniform, linear ∼25% recovery for poly-ubiquitylated proteins (Figure S4D), consistent with prior observations;^11^ therefore, we propose that this mutant should be described as “low-binding” rather than “binding-deficient”.

Finally, the stability and batch effects between trTUBE bead preparations were evaluated, by comparing beads stored for 6 months at 4 °C with a freshly prepared batch (Figures 2B­(iii) and S4E). Poly-ubiquitylated protein IDs, diGly IDs, abundances, and reproducibility were almost identical between batches, indicating the robustness of the Ubi-SCAPE tool and method.

### Ubi-SCAPE Captures Expected Poly-Ubiquitylome Changes with Heat-Shock

To demonstrate the ability of Ubi-SCAPE to capture biologically relevant changes in poly-ubiquitylation, we focused on the confident poly-ubiquitylated proteins that were classified as differentially abundant upon heat-shock, comparing Ubi-SCAPE vs the standard wash buffer with targeted TEVp-assisted elution (Figure 3A). Heat-shock is a well-established stress that leads to a global, proteome-wide increase in poly-ubiquitylation.^22,36^ This directionality of heat-shock-induced changes was far more apparent with our stringent wash conditions and was almost completely lost with the mild washes at the protein level (Figure 3A, 2717 proteins up vs 553 down with stringent washes, compared with 873 up vs 747 down with standard washes). More broadly, reduced background contamination from stringent wash conditions substantially improved the quantitative evaluation of the effects of heat-shock, with over 3 times as many poly-ubiquitylated proteins and diGly peptides quantified as increased in abundance in response to stress (2717 vs 873 and 981 vs 326, respectively).

**Figure 3:**
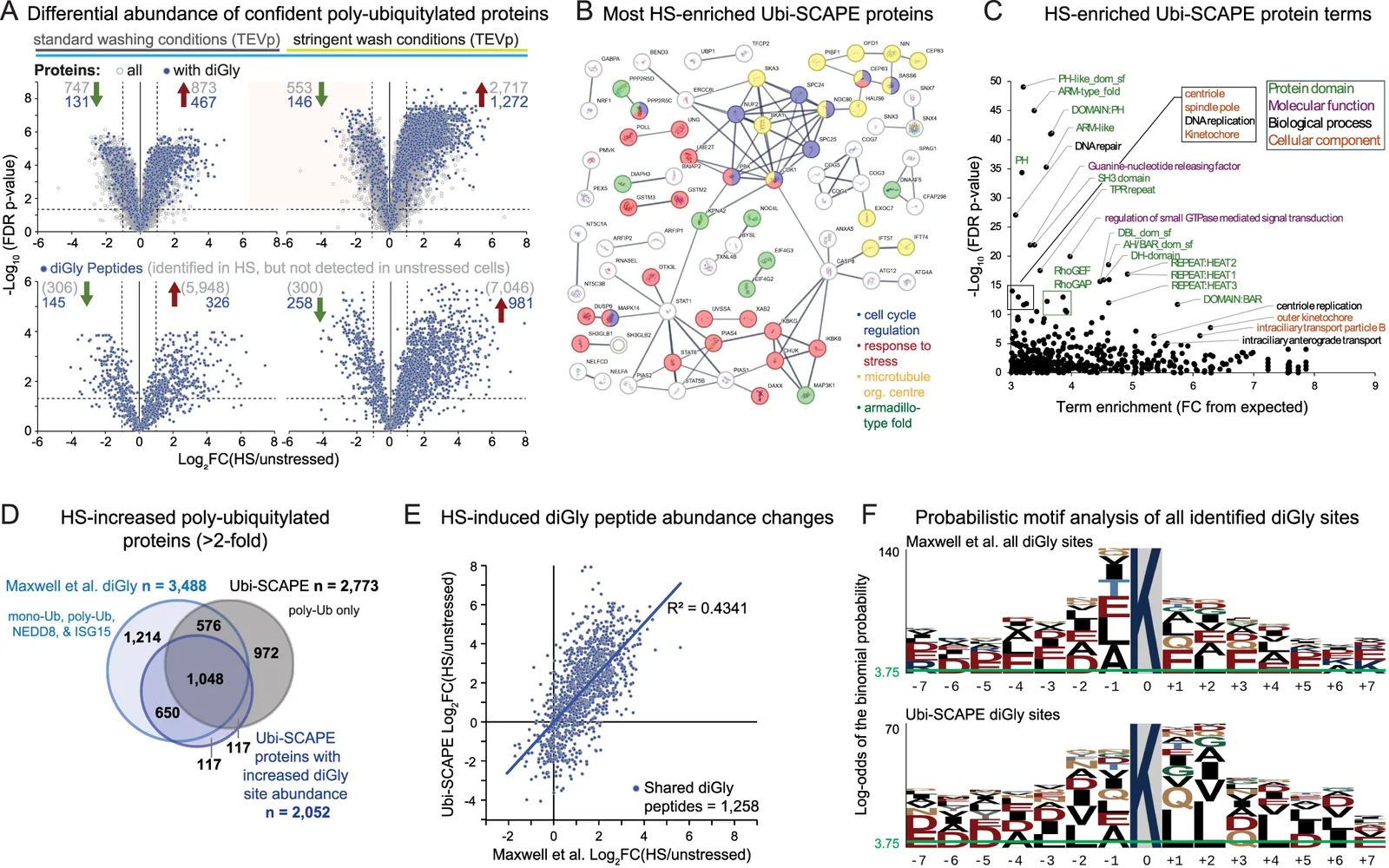
Ubi-SCAPE enables similar biological insights into the heat-shock-induced poly-ubiquitylome as more resource-intensive peptide-level diGly enrichment. (A) Heat-shock-induced changes to abundance of poly-ubiquitylated proteins (with those observed with diGly sites in blue) and diGly peptides, from the experiment described in Figure 2, with the standard vs stringent washing conditions. (B) StringDB network of the top 200 significantly overabundant poly-ubiquitylated proteins with heat-shock, trimmed of unconnected proteins. Confidence >0.7. (C) Descriptive gene term enrichment for the 2717 poly-ubiquitylated proteins that increase upon heat-shock. (D) Proteins observed with increased ubiquitylation (and ubiquitin-like modifiers) upon heat-shock by Maxwell et al.^22^ using diGly isolation vs the poly-ubiquitin-specific Ubi-SCAPE. (E) The correlation between diGly peptide abundance changes upon heat-shock identified by Maxwell et al.^22^ and Ubi-SCAPE. (F) pLOGO probabilistic motif analysis for sites of poly-ubiquitylation observed by Ubi-SCAPE, alongside a data set using an anti-diGly antibody to enrich for all diGly peptides (Maxwell et al.,[Bibr ref22] 2020), both from heat-shocked HEK293 cells.

Exploration of the gene term enrichment emerging from these 2717 heat-shock-increased Ubi-SCAPE proteins (Table S4, and Figure S5) identified well-established signatures of heat-shock (Figure 3B,C).^22,36^ Furthermore, analysis of enriched protein domain terms indicated several domains that are typically present in tandem repeats (e.g., ARM, HEAT, TPR), which would be more prone to stress-induced aggregation,^37^ and/or feature in molecular chaperones that recognize such heat-aggregating proteins (Figure 3C).^38,39^

Finally, we benchmarked our observations with the Maxwell et al.^22^ data set, which used the same cell line and stress (although milder: 43 °C for 1 h). Their initial ubiquitylomics experiments with a HaloTag–4×TUBE enriched ∼300–1000 proteins total (depending on the experiment); therefore, they performed their in-depth analyses using diGly-antibody-based peptide isolation. When we compared the diGly peptides captured from our protein-level Ubi-SCAPE workflow with their more resource-intensive diGly-peptide-based enrichment approach, we found that 65% (2310) of the proteins observed with over 2-fold increased ubiquitylation upon heat-shock by diGly peptide isolation were also profiled as such by Ubi-SCAPE, with a further 1,206 unique to Ubi-SCAPE, and 1,214 unique to Maxwell et al.’s^22^ diGly data set (Figure 3D). Furthermore, for those diGly peptides quantified in both experiments, a correlation (*R* ^2^ = 0.43) and a consistent trend emerged between the two methods (Figure 3E).

As diGly peptide isolation additionally captures mono-ubiquitylation and some ubiquitin-like modifiers, noteworthy differences were anticipated in the motifs around the ubiquitylated lysine for the diGly peptides identified by the two methods. Surprisingly, there were strong similarities between the motifs, although Ubi-SCAPE exhibited a slight preference for the adjacency of the modified lysine to small hydrophobic amino acids (Figure 3F).

In summary, the Ubi-SCAPE protocol offers high-confidence poly-ubiquitylated protein purification, with low variance, high reproducibility, and high purity, thus providing a robust development toward comprehensive poly-ubiquitylomics.

## Conclusions

In this study, we introduce a substantial progression in the application of poly-ubiquitin enrichment tools, with Ubi-SCAPE providing greater sensitivity and specificity for the robust and reproducible isolation of over 7800 poly-ubiquitylated proteins and 8500 diGly peptides. By combining optimized washing and enzymatic elution, we adapted a streamlined version of trTUBE-based purification for seamless integration into high-throughput and/or high-sensitivity LC-MS proteomics workflows, reducing contamination on non-binding beads to just 2.4% of that observed with the previous workflow. Compared with the more time-, cost-, and input-material-intensive ubiquitin remnant motif antibody-based peptide isolation, Ubi-SCAPE demonstrated similar biological insights, in addition to greater specificity toward poly-ubiquitylation, (i.e., reducing signal from mono- and di-ubiquitylated proteins, and diGly remnants from other ubiquitin-like modifiers such as NEDD8 and ISG15).^6^ Although Ubi-SCAPE likely also isolates some proteins exhibiting considerable multi-mono-ubiquitylation, and loses linkage-specific information during digestion, the method offers a means to heavily enrich poly-ubiquitylated proteins, deplete mono-ubiquitylated proteins and contaminants, and provide deeper global insights into the complex and diverse nature of ubiquitylation.

Overall, Ubi-SCAPE offers an optimized, streamlined, and highly reproducible protocol for the characterization of the poly-ubiquitylated proteome to previously unseen depths. The pipeline promises to transform accessibility of the poly-ubiquitylome to researchers, opening new avenues of investigation and understanding of this highly impactful yet understudied PTM.

## Supplementary Material





## Data Availability

The MS proteomics data have been deposited to the ProteomeXchange Consortium (http://proteomecentral.proteomexchange.org) via the PRIDE partner repository with the data set identifier PXD073597.

## Notes

Evaluation of magnetic HaloTag bead saturation with *E. coli* lysate expressing HaloTag–TEVc–6×trTUBE (Figure S1); initial evaluation of tolerated binding conditions for poly ubiquitin chains with HaloTag–TEVc–6×trTUBE (Figure S2); further evaluation of binding conditions of HaloTag–TEVc–6×trTUBE to varying lengths of poly-ubiquitin chains (Figure S3); proteomics evaluation of trTUBE variants and buffers (Figure S4); top 6 enriched terms per DAVID category among the Ubi-SCAPE heat-shock-enriched proteome (Figure S5) (PDF)
The protein group IDs for the trTUBE and control experiments (Table S1); ubiquitin remnant motif peptide IDs (Table S2); MS2-diaPASEF IM-*m*/*z* window definition (Table S3); DAVID–generated terms list for proteins with significantly increased poly-ubiquitylation upon heat-shock (Table S4); key resources table (Table S5) (XLSX)

## ⊥

Cambridge Institute for Medical Research, University of Cambridge, Cambridge CB2 0XY, UK

## Abbreviations

Six×His: hexa-histidine affinity tag
BCA: bicinchoninic acid
diGly: Lys-ε-GlyGly ubiquitin tryptic remnant motif
HS: heat-shock
IP: immunoprecipitation
PMSF: phenylmethylsulfonyl fluoride
polyUb: poly ubiquitin
PTM: post-translational modification
rpm: revolutions per minute
SDS: sodium dodecyl sulfate
SDS-PAGE: sodium dodecyl sulfate-based polyacrylamide gel electrophoresis
SPD: samples-per-day
SPE: solid-phase extraction
TCEP: tris­(2-carboxyethyl)­phosphine
TEVc: Tobacco Etch Virus protease cleavage sequence (ENFLQG)
TEVp: Tobacco Etch Virus protease
TFA: trifluoroacetic acid
trTUBE: trypsin-resistant tandem ubiquitin binding entity
Ub: ubiquitin
Ubi-SCAPE: Ubiquitylomics by Stringent, Cleavable, Affinity-based Proteome Extraction
